# Association Between Heart Rate and Major Adverse Cardiovascular Events Among 9,991 Hypertentive Patients: A Multicenter Retrospective Follow-Up Study

**DOI:** 10.3389/fcvm.2021.741784

**Published:** 2021-12-03

**Authors:** Ningling Sun, Yuanyuan Chen, Yang Xi, Hongyi Wang, Luyan Wang

**Affiliations:** Department of Hypertension, Heart Center, Peking University People's Hospital, Beijing, China

**Keywords:** hypertension, major adverse cardiovascular events, multicenter study, Chinese, heart rate

## Abstract

**Objective:** To assess the effect of heart rate at baseline on major adverse cardiovascular events (MACEs) among hypertensive patients in China.

**Methods:** A multicenter retrospective study was conducted with a 24 month follow-up period. A total of 10,031 hypertensive patients treated with standard antihypertensive drugs were grouped according to their heart rate before treatment: <65 beats per min (bpm), 65–69 bpm, 70–74 bpm, 75–79 bpm, and ≥80 bpm. The occurrence of any of MACEs was as the endpoint event during the 24 month follow-up period. The effect of heart rate at baseline on MACEs was analyzed using univate and multivariable Cox proportional regression analyses, with hazard ratios (HRs) and 95% confidence intervals (CIs). The restricted cubic spline (RCS) model was used to fit the Cox proportional harzard model with 5 knots at the 5^th^, 25^th^, 50^th^, 75^th^, and 95^th^ percentiles of heart rate.

**Results:** Totally 9,991 patients were finally enrolled with the mean systolic pressure (SBP)/diastolic pressure (DBP) of 130.59 ± 7.13/77.66 ± 5.99 mmHg at 24 month follow-up. The incidence of MACEs was 4.80% (*n* = 480). After adjustment for age, gender, baseline blood pressure, alcohol drinking, smoking, hyperlipidemia, diabetes, coronary heart disease, cerebrovascular disease and antihypertensive drug use, patients with heart rate <65 bpm (HR = 1.450, 95% CI: 1.098–1.915) and ≥80 bpm (HR = 1.391, 95% CI: 1.056–11.832) showed 0.45 fold and 0.391 fold increases of MACE risks, compared with patients with heart rate of 70–74 bpm. Furthermore, MACE risks were increased by 86.0% and 65.4% in men, and 59.3% and 69.0% in elderly patients aged ≥65 years at heart rate <65 bpm or ≥80 bpm, respectively. We also found a non-liner U-shaped correlation between heart rate and the occurrence of MACEs.

**Conclusions:** Heart rate might be an independent risk factor for MACEs in hypertensive patients. An appropriate range of heart rate control may offer guidance to hypertension treatment.

## Introduction

Hypertension is a chronic disease with a highest prevalence in China, which is a serious threat to the life and health of the Chinese population (1). It is estimated that approximately 245 million Chinese adults suffer from hypertension in 2019 (2). Previous studies showed that hypertension is a growing burden of cardiovascular disease, and about half of cardiovascular deaths each year are related to hypertension (3). Identification of influencing factors of cardiovascular prognoses in hypertensive patients is essential to help clinicians to assess the disease risk and guide corresponding treatments (4–6).

Resting heart rate is an easy-to-measure phenotypic quantitative trait that is routinely used for risk prediction in the field of cardiovascular medicine (7, 8). Several studies have shown that heart rate is associated with the risk of cardiovascular events and mortality in hypertensive patients (5, 6). After adjusting other risk factors such as diabetes, obesity, and waist-length, elevated resting heart rate is still an independent risk factor for cardiovascular disease (9). However, there are limited studies on the magnitude of heart rate elevation and various definitions of high heart rate in patients with hypertension.

Herein, we investigated the association between heart rate at baseline and the risk of major adverse cardiovascular events (MACEs) in hypertensive patients, and further explored an appropriate range of heart rate control which may improve cardiovascular prognoses in hypertensive patients.

## Materials and Methods

### Study Design and Population

This was a multicenter, retrospective, and follow-up study. All hypertensive patients treated with levoamlodipine maleate or amlodipine besylate at 110 centers in 21 cities in China, came from a previous study (10), and met the standards of the Chinese Guidelines for Prevention and Treatment of Hypertension (4). All the patients received calcium channel blockers (CCBs) levamlodipine maleate or amlodipine besylate, and were followed up for further 24 months. If the target blood pressure reduction was not achieved (<140/90 mmHg), other antihypertensive drugs can be added until the blood pressure was <140/90 mmHg. According to resting heart rate (beats per minute, bpm), the enrolled patients were categorized as five groups: <65, 65–69, 70–74, 75–79, and ≥80 bpm. All the patients signed informed consent forms and this study was approved by human ethics committees with registration number NCT01844570.

### Study Variables

#### Outcome Variables

MACEs as the endpoint of this study were defined as any of the following events occurred during the 24 month follow-up period, including death, non-fatal stroke, non-fatal myocardial infarction, unstable angina pectoris, coronary intervention, coronary artery bypass grafting, new onset of atrial fibrillation, heart failure, or aortic dissection aneurysm. An independent Clinical Event Committee whose members were blinded to the grouping of patients during the study made a judgment on of MACEs.

#### Independent Variables

Blood pressure was measured by an Omron (model: hem-8102a) medical automatic electronic sphygmomanometer in a face-to-face setting in the consulting room. The patients had been told not to engage in intensive exercise half an hour before blood pressure and heart rate measurement. In addition, smoking and coffee were prohibited on the day of the measurement. The resting setting was achieved by sitting for 5 min, the patient's legs were straight and relaxed, and their right arm was exposed and placed on the level of the heart. The heart rate (bpm), blood pressure (mmHg), systolic blood pressure (SBP), and diastolic blood pressure (DBP) of the patients were detected by an automatic digital blood pressure meter. Each index was measured twice and the average value was recorded as the final reading.

#### Covariates

Patients demographics and clinical data were collected, including age, gender, height, weight, alcohol drinking history, smoking history, medical history (hyperlipidemia, diabetes, cardiovascular and cerebrovascular diseases), family history (hypertension, diabetes, stroke, coronary heart disease), drug use [CCBs, renin-angiotensin system inhibitors (RASIs), adrenergic beta blockers, diuretics, and other drugs], etc.

### Follow-Up

The follow-up time of each hypertensive patient was 24 months in the study. Patients were followed up once a month in the first 6 months and once every 3 months thereafter at outpatient department of the hospital. The positive event during 24 month follow-up was MACEs, and the follow-up was terminated when any positive event occurred during the follow-up period. The follow-up sites were located at 110 centers in 21 cities, where the physicians were responsible for the diagnosis and the collection of follow-up information.

### Statistical Analysis

After continuous enrollment, the number of patients who completed the 24 month follow-up was counted and analyzed. All data were tested for normality through the Shapiro test. Comparison between two groups was conducted by the Kruskal-Wallis test. Comparison of means between multiple groups was conducted using one-way ANOVA. The multivariate Cox proportional hazard model was used to identify independent covariates and to estimate the effect of heart rate on the risk of MACEs, with hazard ratios (HRs) and confidence intervals (CIs). Variables achieving *P*-value <0.10 were entered into multivariate analysis during stepwise iteration. The cumulative incidence hazards of MACEs in different heart rate groups were assessed using the Kaplan-Meier method. The restricted cubic spline (RCS) model was used to fit the Cox proportional hazard model with 5 knots at the 5^th^, 25^th^, 50^th^, 75^th^, and 95^th^ percentiles of heart rate. All statistical analyses were performed using SAS 9.4 (SAS Institute, Cary, NY, USA). *P* value <0.05 was considered statistically different.

## Results

### Characteristics of Patients

**Figure 1:**
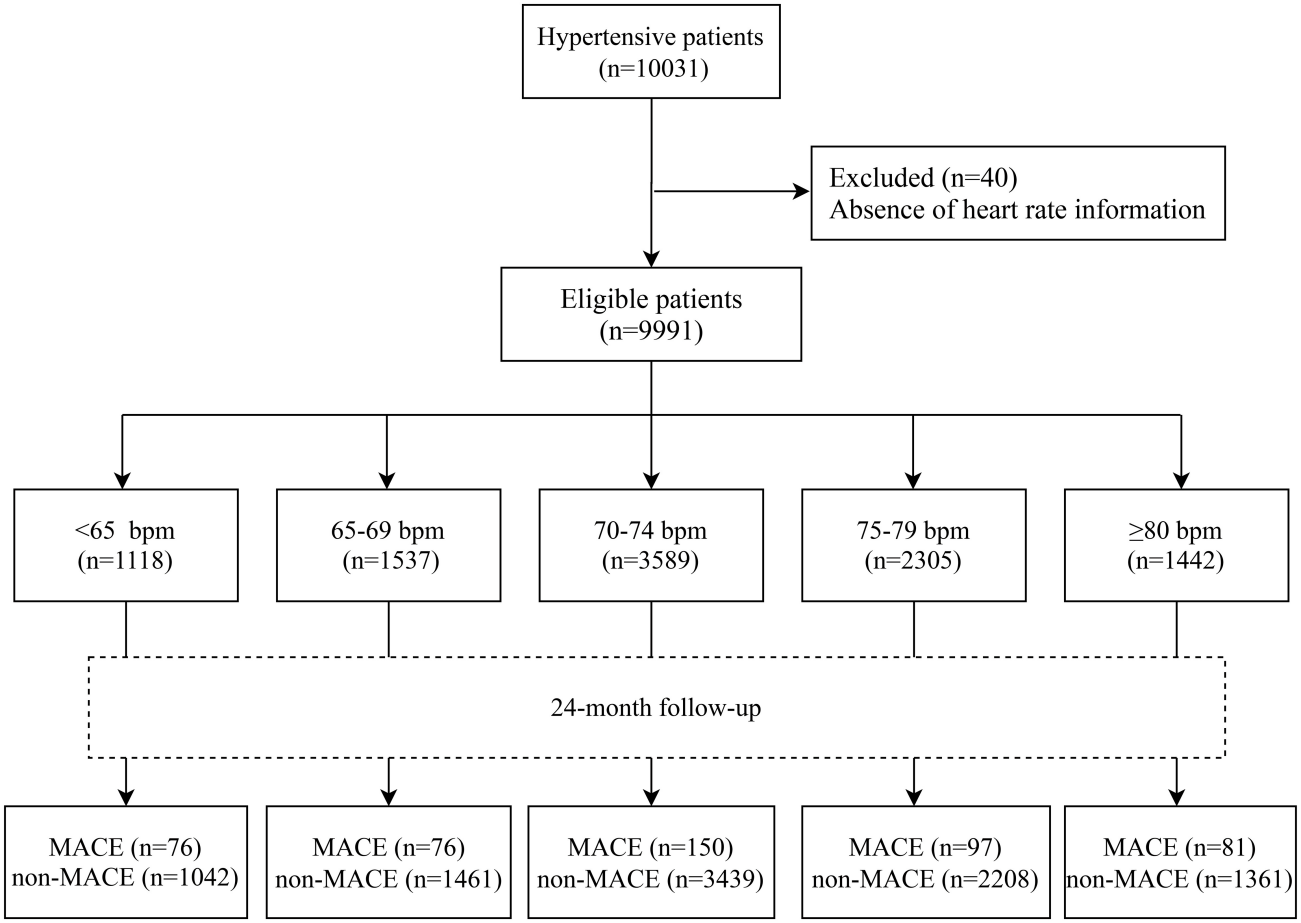
Flow chart of patient screening.

Figure 1 showed the flow chart of patient screening. A total of 10,031 patients with hypertension were enrolled, and 40 patients without heart rate information were excluded. Then 9,991 hypertensive patients were finally included in this study, with the mean age of 64.46 ± 10.65 years, the mean BMI of 24.61 ± 2.82 kg/m^2^, the mean SBP of 145.43 ± 17.41 mmHg, and the mean DBP of 84.90 ± 10.67 mmHg. Baseline data on age, sex, blood pressure, and BMI exhibited no statistical differences between the 40 excluded and 9,991 included. We found that there were significant differences between the five groups of heart rate levels in age (*F* = 22.588, *P* < 0.001), gender (χ^2^ = 10.878, *P* = 0.028), BMI (*F* = 2.678, *P* = 0.030), baseline SBP (*F* = 90.108, *P* < 0.001), baseline DBP (*F* = 95.214, *P* < 0.001), alcohol drinking (χ^2^ = 22.329, *P* < 0.001), smoking (χ^2^ = 34.525, *P* < 0.001), medical history (coronary heart disease: χ^2^ = 134.268, *P* < 0.001; cerebrovascular disease: χ^2^ = 34.975, *P* < 0.001), family history (hypertension: χ^2^ = 134.268, *P* = 0.029; coronary heart disease: χ^2^ = 134.268, *P* = 0.005), and drug use (CCBs: χ^2^ = 10.439, *P* = 0.034; RASIs: χ^2^ = 17.266, *P* = 0.002; adrenergic beta blockers: χ^2^ = 64.223, *P* < 0.001; diuretics: χ^2^ = 24.567, *P* < 0.001; other drugs: χ^2^ = 12.419, *P* = 0.014). The incidences of MACEs in the five groups were found to be significantly different (χ^2^ = 16.719, *P* = 0.002). Demographic data, history of diseases, drug use and outcomes of hypertension cases were shown in Table 1.

**Table 1:** Characteristics of hypertensive patients at different heart rate levels.

**Variables**	**Total (*n* = 9,991)**	**<65 bpm (*n* = 1,118)**	**65–69 bpm (*n* = 1,537)**	**70–74 bpm (*n* = 3,589)**	**75–79 bpm (*n* = 2,305)**	**≥80 bpm (*n* = 1,442)**	**Statistics**	***P-*value**
Age, years, Mean ± SD	64.46 ± 10.65	66.82 ± 10.57	64.95 ± 10.78	64.47 ± 10.47	63.28 ± 10.37	63.94 ± 11.17	*F* = 23.409	<0.001
Gender, *n* (%)							*χ^2^* = 10.878	0.028
Male	5,045 (50.50)	558 (49.91)	726 (47.23)	1,811 (50.46)	1,188 (51.54)	762 (52.84)		
Female	4,946 (49.50)	560 (50.09)	811 (52.77)	1,778 (49.54)	1,117 (48.46)	680 (47.16)		
BMI, kg/m^2^, Mean ± SD	24.61 ± 2.82	24.71 ± 2.88	24.50 ± 2.75	24.53 ± 2.78	24.72 ± 2.78	24.66 ± 2.99	*F* = 2.355	0.070
**BP, mmHg, Mean** **±** **SD**								
SBP	145.43 ± 17.41	144.65 ± 17.71	142.19 ± 16.51	143.59 ± 16.65	146.35 ± 16.83	152.62 ± 18.73	*F* = 115.527	<0.001
DBP	84.90 ± 10.67	82.72 ± 11.27	82.78 ± 10.56	84.13 ± 10.16	85.99 ± 10.10	89.04 ± 11.10	*F* = 126.957	<0.001
Alcohol, *n* (%)	7,332 (73.39)	852 (76.21)	1,174 (76.38)	2,613 (72.81)	1,688 (73.23)	1,005 (69.69)	χ^2^ = 22.329	<0.001
Smoking, *n* (%)	7,443 (74.50)	862 (77.10)	1,210 (78.72)	2,663 (74.20)	1,698 (73.67)	1,010 (70.04)	χ^2^ = 34.525	<0.001
**Present history**, ***n*** **(%)**								
Hyperlipidemia	858 (8.59)	111 (14.29)	139 (13.83)	294 (8.19)	188 (8.16)	126 (8.74)	*χ^2^* = 3.627	0.305
Diabetes	1,477 (14.78)	176 (15.74)	246 (16.01)	507 (14.13)	320 (13.88)	228 (15.81)	*χ^2^* = 6.525	0.089
Coronary heart disease	1,446 (14.47)	264 (23.61)	283 (18.41)	458 (12.76)	240 (10.41)	201 (13.94)	χ^2^ = 134.268	<0.001
Cerebrovascular disease	1,056 (10.57)	173 (15.47)	158 (10.28)	372 (10.37)	208 (9.02)	145 (10.06)	χ^2^ = 34.975	<0.001
**Family history**, ***n*** **(%)**								
Hypertension	3,480 (34.83)	360 (63.38)	524 (65.01)	1,290 (35.94)	792(34.36)	514 (35.64)	*χ^2^* = 5.359	0.147
Stroke	391 (3.91)	54 (11.18)	56 (9.11)	136 (3.79)	81 (3.51)	64 (4.44)	*χ^2^* = 2.553	0.466
Coronary heart disease	594 (13.64)	87 (17.51)	105 (16.30)	194 (12.15)	124 (12.33)	84 (13.77)	χ^2^ = 14.684	0.005
Diabetes	540 (5.40)	61 (12.53)	93 (14.55)	183 (5.10)	121(5.25)	82 (5.69)	*χ^2^* = 1.802	0.614
**Present drug use**, ***n*** **(%)**								
CCBs							*χ^2^* = 10.439	0.034
Levamlodipine maleate	4,994 (49.98)	523 (46.78)	783 (50.94)	1,760 (49.04)	1,199 (52.02)	729 (50.55)		
Amlodipine besylate	4,997 (50.02)	595 (53.22)	754 (49.06)	1,829 (50.96)	1,106 (47.98)	713 (49.45)		
β-blockers							*χ^2^* = 64.223	<0.001
Yes	2,087 (20.89)	280 (25.04)	380 (24.72)	688 (19.17)	389 (16.88)	350 (24.27)		
No	7,904 (79.11)	838 (74.96)	1,157 (75.28)	2,901 (80.83)	1,916 (83.12)	1,092 (75.73)		
RASIs	3,630 (36.33)	451 (40.34)	600 (39.04)	1,262 (35.16)	803 (34.84)	514 (35.64)	*χ^2^* = 17.266	0.002
Diuretics	392 (3.92)	59 (5.28)	53 (3.45)	129 (3.59)	69 (2.99)	82 (5.69)	*χ^2^* = 24.567	<0.001
Other drugs	641 (6.42)	77 (6.89)	105 (6.83)	259 (7.22)	118 (5.12)	82 (5.69)	*χ^2^* = 12.419	0.014
MACEs, *n* (%)							*χ^2^* = 16.719	0.002
No	9,511 (95.20)	1,042 (93.20)	1,461 (95.06)	3,439 (95.82)	2,208 (95.79)	1,361 (94.38)		
Yes	480 (4.80)	76 (6.80)	76 (4.94)	150 (4.18)	97 (4.21)	81 (5.62)		

*SD, standard deviation; BMI, body mass index; SBP, systolic blood pressure; DBP, diastolic blood pressure; CCB, calcium antagonist; RASI, renin angiotensin system inhibitor; MACEs, major adverse cardiovascular events*.

### Influence of Heart Rate on MACEs in All Patients

**Figure 2:**
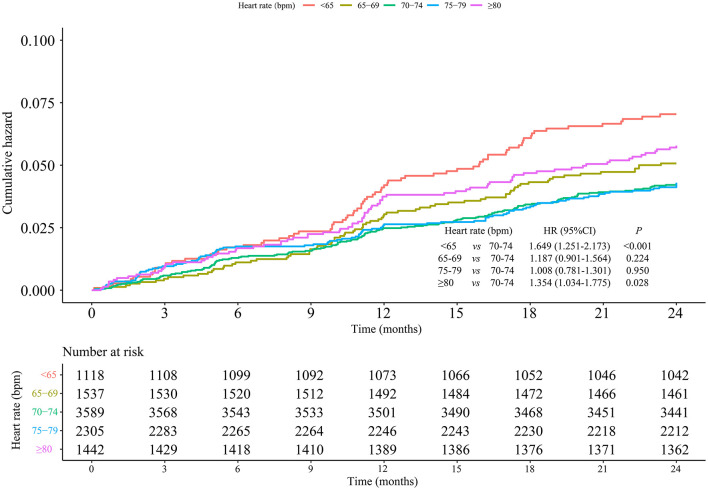
Cumulative incidence of MACEs in different groups of heart rate.

**Figure 3:**
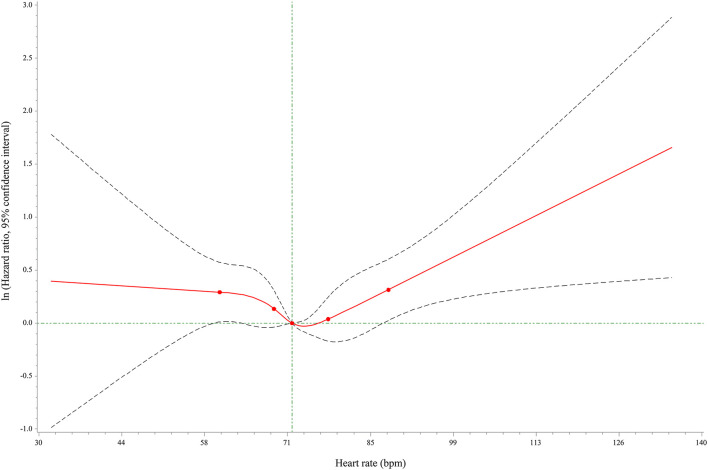
RCS curve for the correlation between heart rate and MACE risks in hypertensive patients.

The incidence of MACEs was 4.80% among 9,991 hypertensive patients. Compared with patients at heart rate of 70–74 bpm, hypertensive patients at heart rate <65 bpm (HR = 1.649, 95% CI: 1.251–2.173) and ≥80 bpm (HR = 1.354, 95% CI: 1.034–1.775) showed higher risks of MACEs. Nevertheless, in contrast to patients with heart rate of 70–74 bpm, those with heart rate of 65–69 and 75–79 bpm showed no differences in MACE risks with HRs of 1.187 (95% CI: 0.901–1.564) and 1.008 (95% CI: 0.781–1.301), respectively. After adjusting for age, gender, baseline blood pressure, alcohol drinking, smoking, hyperlipidemia, diabetes, coronary heart disease, cerebrovascular disease, CCBs, beta-blockers, RASIs, diuretics, and other drugs, there were 0.45 fold and 0.391 fold increases in the risks of MACEs in patients with heart rate <65 bpm (HR = 1.450, 95% CI: 1.098–1.915) and ≥80 bpm (HR = 1.391, 95% CI: 1.056–11.832), while no significant differences were found in the other groups (Table 2). The cumulative incidence hazards of MACEs in different groups were shown in Figure 2. Moreover, the RCS curve displayed that the heart rate of hypertensive patients was in non-linear correlation with the incidence of MACEs (Figure 3). When the heart rate was 72 bpm, the risk of MACEs was the lowest.

**Table 2:** Association between heart rate and MACE risks in all hypertensive patients.

**Heart rate (bpm)**	**HR (95%CI)**	***P-*value**	HR (95%CI)^*^	***P-*value**
<65	1.649 (1.251–2.173)	<0.001	1.450 (1.098–1.915)	0.009
65–69	1.187 (0.901–1.564)	0.224	1.141 (0.865–1.505)	0.865
70–74	Ref		Ref	
75–79	1.008 (0.781–1.301)	0.950	1.094 (0.846–1.415)	0.492
≥80	1.354 (1.034–1.775)	0.028	1.391 (1.056–11.832)	0.019

*HR, hazard ratio; CI: confidence interval*.

* *adjusting for age, gender, baseline blood pressure, driking alcohol, smoking, hyperlipidemia, diabetes, coronary heart disease, cerebrovascular disease, CCBs, beta-blockers, RASIs, diuretics, and other drugs*.

### Association Between Heart Rate and MACEs by Gender and Age

**Figure 4:**
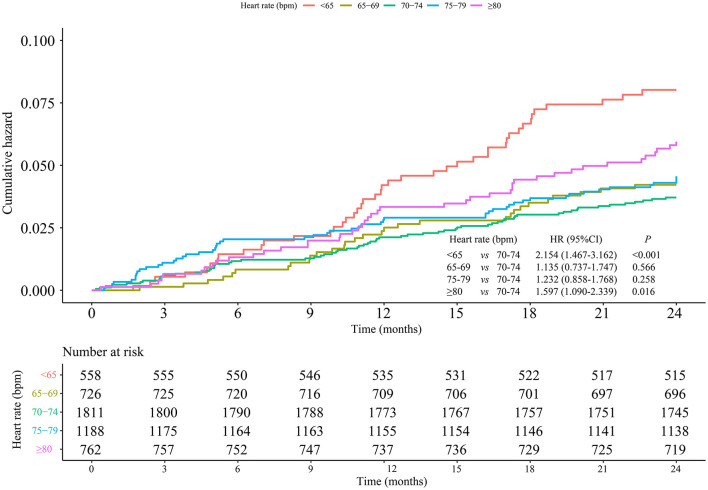
Association between heart rate and MACE risks in hypertensive male patients.

**Figure 5:**
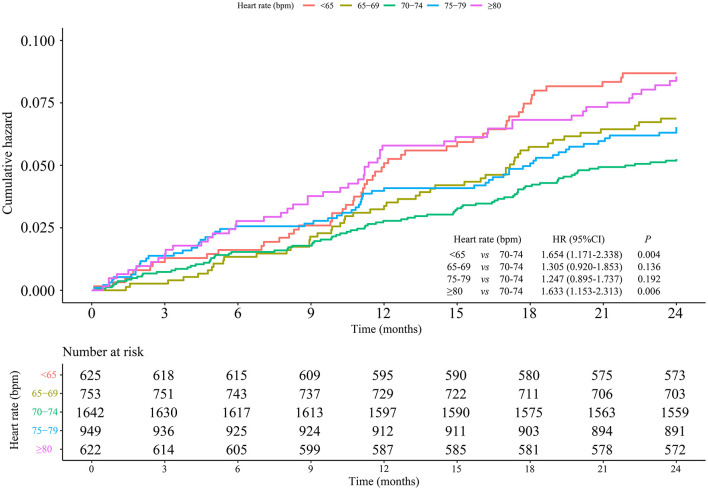
Association between heart rate and MACE risks in hypertensive elderly patients.

After adjustment for age, baseline blood pressure, alcohol drinking, smoking, hyperlipidemia, diabetes, coronary heart disease, cerebrovascular disease, CCBs, beta-blockers, RASIs, diuretics, and other drugs, the results showed that the risks of MACEs were significantly higher in men with heart rate <65 bpm (HR = 1.860, 95% CI: 1.260–2.745) and ≥80 bpm (HR = 1.654, 95% CI: 1.120–2.442) than those with heart rate of 70–74 bpm. Moreover, when gender, baseline blood pressure, alcohol drinking, smoking, hyperlipidemia, diabetes, coronary heart disease, cerebrovascular disease, CCBs, beta-blockers, RASIs, diuretics, and other drugs were adjusted, elderly patients (≥65 years old) at heart rate <65 bpm (HR = 1.593, 95% CI: 1.125–2.255) and ≥80 bpm (HR = 1.690, 95% CI: 1.184–2.412) had significantly increased MACE risks in comparison with those at heart rate of 70–74 bpm, respectively. There was no difference in women and the group aged <65 years old (Table 3). The cumulative incidence hazards of MACEs in male and elderly patients were shown in Figure 4 and Figure 5, respectively.

**Table 3:** Association between heart rate and MACE risks in subgroups of gender and age.

**Subgroups**	**Heart rate (bpm)**	**HR (95%CI)**	***P-*value**	**HR (95%CI)**	***P-*value**
**Male**	<65	2.154 (1.467–3.162)	<0.001	1.860 (1.260–2.745)^a^	0.002
	65–69	1.135 (0.737–1.747)	0.566	1.117 (0.725–1.722)^a^	0.616
	70–74	Ref		Ref	
	75–79	1.232 (0.858–1.768)	0.258	1.330 (0.925–1.912)^a^	0.124
	≥80	1.597 (1.090–2.339)	0.016	1.654 (1.120–2.442)^a^	0.011
**Female**	<65	1.259 (0.842–1.884)	0.262	1.097 (0.731–1.646)^a^	0.656
	65–69	1.206 (0.842–1.728)	0.307	1.117 (0.778–1.605)^a^	0.548
	70–74	Ref		Ref	
	75–79	0.831 (0.577–1.196)	0.319	0.882 (0.610–1.273)^a^	0.502
	≥80	1.161 (0.789–1.709)	0.450	1.135 (0.766–1.683)^a^	0.528
**<65 years**	<65	1.448 (0.908–2.311)	0.120	1.246 (0.776–2.003)^b^	0.362
	65–69	0.979 (0.622–1.541)	0.927	0.924 (0.585–1.461)^b^	0.737
	70–74	Ref		Ref	
	75–79	0.802 (0.536–1.200)	0.284	0.833 (0.556–1.247)^b^	0.374
	≥80	1.079 (0.701–1.661)	0.730	1.046 (0.673–1.625)	0.843
**≥65 years**	<65	1.654 (1.171–2.338)	0.004	1.593 (1.125–2.255)^b^	0.009
	65–69	1.305 (0.920–1.853)	0.136	1.337 (0.959–1.866)^b^	0.087
	70–74	Ref		Ref	
	75–79	1.247 (0.895–1.737)	0.192	1.337 (0.959–1.866)^b^	0.087
	≥80	1.633 (1.153–2.313)	0.006	1.690 (1.184–2.412)^b^	0.004

*HR, hazard ratio; CI: confidence interval*.

a *adjusting for age, baseline blood pressure, driking alcohol, smoking, hyperlipidemia, diabetes, coronary heart disease, cerebrovascular disease, CCBs, beta-blockers, RASIs, diuretics, and other drugs*;

b *adjusting for gender, baseline blood pressure, driking alcohol, smoking, hyperlipidemia, diabetes, coronary heart disease, cerebrovascular disease, CCBs, beta-blockers, RASIs, diuretics, and other drugs*.

## Discussion

In recent years, heart rate management in patients with hypertension has attracted major attention in clinic. However, the range of beneficial heart rate in hypertensive patients is unclear and still under debate (11). This study aimed to assess the optimal heart rate of hypertensive patients by grouping patients with different heart rate levels at baseline before treatment, which may affect the prognoses of hypertensive patients. Our findings showed that heart rate <65 bpm and ≥80 bpm were associated with the high risks of MACEs in hypertensive patients, especially in men and the elderly. In addition, we also found a non-liner U-shaped correlation between heart rate and the occurrence of MACEs, with heart rate of 72 bpm at the lowest risk.

Some studies have revealed that increased heart rate in patients with hypertension was a marker of increased sympathetic excitability (12, 13). The increase of heart rate was a common clinical presentation of hypertension. A survey conducted in France which included 100,000 people reported that the average heart rate of untreated patients with hypertension increased by 6 bpm compared with that of those patients with normotension (14). A cross-sectional study of 115,229 hypertensive patients from 21 cities in China showed that the average resting heart rate of hypertensive patients was 76.6 bpm, and there were a striking 38.2% of uncomplicated hypertensive patients having average resting heart rate ≥80 bpm (15). High heart rate increased the risk of cardiovascular events in patients with hypertension (16). The Kailuan study in an Asian population illustrated that individuals with resting heart rate of >78 bpm had a 16% higher risk of developing hypertension than those <70 bpm after 3.5 years of follow-up (17). Data of 2,293 untreated patients over 60 years old with isolated systolic hypertension from the European Systolic Hypertensive Trial demonstrated that there was an 89% increase in all-cause mortality for patients with baseline heart rate ≥80 bpm (18). Zhao et al. (19) showed that resting heart rate >79 bpm elevated the risk of heart failure by 97% compared with that ≤ 69 bpm. Zhang et al. (20) reported that increased heart rate related to the increase of cardiovascular deaths to a certain extent. In a South Korean cohort study, 6,100 community residents were followed up for 20.8 years and higher resting heart rate and hypertension were found to synergistically increase future cardiovascular events (21). Compared with normotensive patients with heart rate of 61–79 bpm, hypertensive patients with higher heart rate ≥80 bpm had a high risk ratio for cardiovascular death, and the ratio for males was 8.34 and the ratio for females was 5.95 (21). Chinese Center for Disease Control and Prevention (China CDC) Weekly report showed that the incidence of cardiovascular disease in hypertensive patients with baseline heart rate of >80 bpm was 1.35 times that in individuals with normal blood pressure (22). Herein, we discovered that heart rate ≥80 bpm in hypertensive patients brought a high risk of MACEs, which was supported by previous studies.

This could be attributed to sympathetic nerve activation, peripheral vascular resistance, and endothelium function (23, 24). Catecholamine levels in the blood of hypertensive patients are higher. An increase in resting heart rate is caused by the increase of catecholamine in the blood (25). It has also been shown that high heart rate increases renal tubular reabsorption of sodium and decreases glomerular filtration rate. Increasing the levels of angiotensin II and renin-angiotensin receptors could lead to a persistent increase in blood pressure, and high blood pressure in patients is associated with the overall cardiac norepinephrine spillage. The feedback loop of cardiac norepinephrine spillage could cause high heart rate (*r* = 0.82, *P* = 9.3 ×10^−5^). Therefore, increased heart rate is a sign of increased cardiac sympathetic activity in patients with hypertension (12, 13). Increased sympathetic tone is found in about 30% of hypertension patients. This abnormality is closely associated with the metabolic syndrome of dyslipidemia and hyperinsulinaemia (26). Early studies have reported that sympathetic stimulation enhances cardiac and vascular hypertrophy, and left ventricular hypertrophy is a strong predictor of poor cardiovascular outcomes. Hypertrophy of resistance vessels accelerates hypertension, whereas hypertrophy of smaller coronary vessels limits coronary reserve and increases a tendency toward coronary spasms. Epidemiologically, high haematocrit is associated with hypertension and is recognized as an independent coronary risk factor. Sympathetic stimulation increases haematocrit through an increase of post-capillary vascular resistance. In addition, sympathetic over-activity is also relevant to platelet activation which may further add to the risk of coronary thrombosis in neurogenic hypertension. High heart rate could cause direct damage to the arteries and promote arrhythmia by hemodynamic force (27, 28). Elevated heart rate could increase hemodynamic stress and shorten the diastolic phase, which could then increase tensile stress, low and oscillatory shear stress, thereby promoting oxygen consumption. These direct detrimental effects could cause coronary atherosclerosis and myocardial ischemia (29).

Furthermore, the result also showed heart rate <65 bpm was related to the occurrence of MACEs among hypertensive cases. A study of middle-aged and older people in China found that low baseline heart rate (<65 bpm) had a higher risk of cardiovascular disease (30). It was indicated that a heart rate reduction was associated with the incidence of cardiovascular disease. Reduced heart rate may lead to dispersed atrial repolarization, and further trigger cardiovascular events (31).

In this current study, we found that the risks of MACEs in male hypertensive patients or hypertensive patients ≥65 years old in the group of heart rate <65 and ≥80 bpm were higher than the risk in the group with heart rate of 70–74 bpm, suggesting the incidence of MACEs was correlated with heart rate of <65 and ≥80 bpm after 24 month follow-up. There were 45.0 and 39.1% increases of MACE risks in the groups of heart rate <65 and ≥80 bpm among all the patients with hypertension, respectively. Moreover, MACE risks were observed to significantly increase to 86.0% (heart rate <65 bpm) and 65.4% (heart rate ≥80 bpm) in the male group, and to 59.3% (heart rate <65 bpm) and 69.0% (heart rate ≥80 bpm) in the elderly group (≥65 years), respectively. It was indicated that managing heart rate at 65–79 bpm may be beneficial in reducing the risk of MACEs in hypertensive cases, particularly in males and the elderly. The gender and age of hypertensive patients have offered prognostic values for cardiovascular diseases (32, 33). Continuous monitoring and management of heart rate may potentially improve the outcomes of hypertensive patients, especially in male and elderly patients.

Interestingly, a non-liner correlation between heart rate and the occurrence of MACEs in a “U” shape was also discovered in this study. An appropriate range of heart rate was 65–79 bpm, which may contribute to minimize the risk of MACEs. Besides, the lowest risk of MACEs was showed at the heart rate of 72 bpm. Current studies suggested that heart rate control (<80 bpm) was effective in the treatment of fast heart rate disorders, such as atrial fibrillation and congestive heart failure, which was similar to our findings. We provide an optimal value of heart rate that may be helpful to keep a steady circadian rhythm of heart rate, and to decrease the risk of MACEs in hypertensive patients. Tachycardia in hypertension is characterized by increased sympathetic tone and decreased vagal activity, and then antihypertensive drugs that can decrease heart rate via reducing sympathetic outflow should be considered as preferential agents. Several personal digital devices including mobile phones, fitness trackers and eHealth applications can be used for monitoring the heart rate of hypertensive patients to help clinicians as well as the public identify the risk.

We conducted a multicenter, retrospective, and follow-up study based on a large Chinese population to assess the association between heart rate and MACE risks in patients with hypertension. The appropriate range and the optimal value of heart rate were mentioned for Chinese hypertensive individuals, which may be useful to guide clinicians in the treatment of hypertension. There are some limitations that should be noted in interpreting our findings. First, this study was based on a previous study “Pragmatic comparative effectiveness trial in a real-world setting,” data about heart rate in the observation period or stable status were not collected, and data on blood glucose and other blood biochemistry indicators could not be monitored. Although most covariables were adjusted, several potential residual confounders may not be controlled (34–36). A propensity score matching method may be useful in future studies. Second, the association between heart rate and MACE risks in patients with hypertension was explored in this study, while individuals with prehypertension were ignored. A Lancet study has reported that the conversion of prehypertension to hypertension which frequently occurs over a period of 4 years is up to 30%, especially in the elderly (37). Prehypertension is also associated with an increased risk of MACEs (38). Whether the optimum heart rate is suitable for patients with prehypertension needs to be determined, which would help to better monitor the heart rate changes of people with different blood pressure levels. Third, the follow-up period of this study was only 24 months, although the results of the study showed the appropriate range of heart rate in hypertensive patients. Well-designed, and longer follow-up studies are still needed to confirm our results.

## Conclusion

In the present study, the relationship between heart rate and MACE risks was investigated among hypertensive patients. The results showed that heart rate of <65 or ≥80 bpm was an independent risk factor for the occurrence of MACEs. The clinical evidence was provided that the heart rate of hypertensive patients was better controlled at 65–79 bpm. Hypertensive patients need to pay attention to the changes in heart rate while strictly controlling their blood pressure. Heart rate can be used as an independent marker of MACEs, which emphasizes the necessity of heart rate management in addition to current treatment regimes in preventing cardiovascular disease.

## Data Availability Statement

The original contributions presented in the study are included in the article/supplementary material, further inquiries can be directed to the corresponding authors.

## Ethics Statement

The studies involving human participants were reviewed and approved by the Ethics Committees of Peking University First Hospital. The patients/participants provided their written informed consent to participate in this study.

## Author Contributions

The overall study design and scheme, medical advice, and article writing came from NS. Data collection and analysis were done by YC, HW, YX, and LW. All authors have read and approved the final manuscript.

## Funding

This study was supported by the National Science and Technology Major Project (No. 2021ZX09101101).

## Conflict of Interest

The authors declare that the research was conducted in the absence of any commercial or financial relationships that could be construed as a potential conflict of interest.

## Publisher's Note

All claims expressed in this article are solely those of the authors and do not necessarily represent those of their affiliated organizations, or those of the publisher, the editors and the reviewers. Any product that may be evaluated in this article, or claim that may be made by its manufacturer, is not guaranteed or endorsed by the publisher.

## References

[1] Chinese Medical Association, Chinese Medical Journals Publishing House, Chinese Society of General Practice, Editorial Board of Chinese Journal of General Practitioners of Chinese Medical Association, Expert Group of Guidelines for Primary Care of Cardiovascular Disease. Guideline for primary care of hypertension (2019). Chin J Gen Pract. (2019) 18:301–13. 10.3760/cma.j.issn.1671-7368.2019.04.002

[2] Hu S. Report on cardiovascular health and diseases in China 2019: updated summary. Chin Circ J. (2020) 35:833–54.

[3] Chen WW Gao RL Liu LS Zhu ML Wang W Wang YJ. China cardiovascular diseases report 2015: a summary. J Geriatr Cardiol. (2017) 14:1–10. 10.11909/j.issn.1671-5411.2017.01.012 28270835 PMC5329726

[4] Li–Sheng LIU Joint Committee for Guideline Revision. 2018 Chinese guidelines for prevention and treatment of hypertension—a report of the revision committee of Chinese guidelines for prevention and treatment of hypertension. J Geriatr Cardiol. (2019) 16:182–241. 10.11909/j.issn.1671-5411.2019.03.014 31080465 PMC6500570

[5] Huang Y Dai M Deng Z Huang X Li H Bai Y. Clustering of risk factors and the risk of new-onset hypertension defined by the 2017 ACC/AHA hypertension guideline. J Hum Hypertens. (2020) 34:372–7. 10.1038/s41371-019-0232-9 31431682

[6] Huang Y Deng Z Se Z Bai Y Yan C Zhan Q. Combined impact of risk factors on the subsequent development of hypertension. J Hypertens. (2019) 37:696–701. 10.1097/HJH.0000000000001956 30817449

[7] Archangelidi O Pujades-Rodriguez M Timmis A Jouven X Denaxas S Hemingway H. Clinically recorded heart rate and incidence of 12 coronary, cardiac, cerebrovascular and peripheralarterial diseases in 233,970 men and women: a linked electronic health record study. Eur J Prev Cardiol. (2018) 25:1485–95. 10.1177/2047487318785228 29966429

[8] Xhaard C Dandine-Roulland C Villemereuil P Floch EL Bacq-Daian D Machu JL. Heritability of a resting heart rate in a 20-year follow-up family cohort with GWAS data: insights from the STANISLAS cohort. Eur J Prev Cardiol. (2021) 28:1334–41. 10.1177/2047487319890763 34647585

[9] Palatini P Rosei EA Casiglia E Chalmers J Ferrari R Grassi G. Management of the hypertensive patient with elevated heart rate: statement of the second consensus conference endorsed by the European society of hypertension. J Hypertens. (2016) 34:813–21. 10.1097/HJH.0000000000000865 26982382

[10] Ma W Sun N Duan C Zhao L Hua Q Sun Y. Effectiveness of levoamlodipine maleate for hypertension compared with amlodipine besylate: a pragmatic comparative effectiveness study. Cardiovasc Drugs Ther. (2021) 35:41–50. 10.1007/s10557-020-07054-1 32915349

[11] Shi ZW Feng YQ Lin JX Chu SL Lu YX Lu XZ. Chinese expert consensus on heart rate management in hypertensive patients (Expert consensus). Chin J Frontiers Med Sci. (2017) 9:29–36.

[12] Esler M Lambert G Esler D Ika Sari C Guo L Jennings G. Evaluation of elevated heart rate as a sympathetic nervous systembiomarker in essential hypertension. J Hypertens. (2020) 38:1488–95. 10.1097/HJH.0000000000002407 32195820

[13] Grassi G Quarti-Trevano F Seravalle G Dell'Oro R Facchetti R Mancia G. Association between the European Society of Cardiology/European Society of Hypertension heart rate thresholds for cardiovascular risk and neuroadrenergic markers. Hypertension. (2020) 76:577–82. 10.1161/HYPERTENSIONAHA.120.14804 32594806

[14] Morcet JF Safar M Thomas F Guize L Benetos A. Associations between heart rate and other risk factors in a large French population. J Hypertens. (1999) 17:1671–6. 10.1097/00004872-199917120-00003 10658932

[15] Sun N Huo Y Huang J. The current status of heart rate in Chinese hypertensive patients. Chin J Hypertens. (2015) 23:934–9. 10.16439/j.cnki.1673-7245.2015.10.013

[16] Lonn EM Rambihar S Gao P Custodis FF Sliwa K Teo KK. Heart rate is associated with increased risk of major cardiovascular events, cardiovascular and all-cause death in patients with stable chronic cardiovascular disease-an analysis of ONTARGET/TRANSCEND. Clin Res Cardiol. (2014) 103:149–59. 10.1007/s00392-013-0644-4 24356937

[17] Wang A Liu X Guo X Dong Y Wu Y Huang Z. Resting heart rate and risk of hypertension: results of the Kailuan cohort study. J Hypertens. (2014) 32:1600–5. 10.1097/HJH.0000000000000230 24879491

[18] Palatini P Thijs L Staessen JA Fagard RH Bulpitt CJ Clement DL. Predictive value of clinic and ambulatory heart rate for mortality in elderly subjects with systolic hypertension. Arch Intern Med. (2002) 162:2313–21. 10.1001/archinte.162.20.2313 12418945

[19] Zhao M Chen Y Wang M Wang C Yao S Li Y. Relationship between resting heart rate and incident heart failure in patients with hypertension: the kailuan cohort study in China. J Clin Hypertens. (2020) 22:2325–31. 10.1111/jch.14062 33017515 PMC8029903

[20] Zhang D Shen X Qi X. Resting heart rate and all-cause and cardiovascularmortality in the general population:a meta-analysis. CAMJ. (2016) 188:E53–63. 10.1503/cmaj.150535 26598376 PMC4754196

[21] Ryu M Bayasgalan G Kimm H Nam CM Ohrr H. Association of resting heart rate and hypertension stages on all-cause and cardiovascular mortality among elderly Koreans: the kangwha cohort study. J Geriatr Cardiol. (2016) 13:573–9. 27605937 10.11909/j.issn.1671-5411.2016.07.003 PMC4996831

[22] Zhang X Zhang M Li C Wang L Wu J Huang Z. Associations between hypertension status and increased heart rate-China, 2015. China CDC Wkly. (2020) 2:771–4. 10.46234/ccdcw2020.209 34594764 PMC8393030

[23] Custodis F Schirmer SH Baumhäkel M Heusch G Böhm M Laufs U. Vascular pathophysiology in response to increased heart rate. J Am Coll Cardiol. (2010) 56:1973–83. 10.1016/j.jacc.2010.09.014 21126638

[24] Lahiri MK Kannankeril PJ Goldberger JJ. Assessment of autonomic function in cardiovascular disease: physiological basis and prognostic implications. J Am Coll Cardiol. (2008) 51:1725–33. 10.1016/j.jacc.2008.01.038 18452777

[25] Tochikubo O Mizushima S Watanabe J Minamisawa K. Base heart rate during sleep in hypertensive and normotensive subjects. J Hypertens. (2001) 19:1131–7. 10.1097/00004872-200106000-00019 11403363

[26] Palatini P. Heart rate and the cardiometabolic risk. Curr Hypertens Rep. (2013) 15:253–9. 10.1007/s11906-013-0342-7 23645136

[27] Tadic M Cuspidi C Grassi G. Heart rate as a predictor of cardiovascular risk. Eur J Clin Invest. (2018) 48:1–11. 10.1111/eci.12892 29355923

[28] Seravalle G Quarti Trevano F Grassi G. Heart rate as a predictor of cardiovascular risk. Minerva Med. (2021) 112:130–43. 10.23736/S0026-4806.20.06695-1 32512980

[29] Palatini P. Elevated heart rate in cardiovascular diseases: a target for treatment? Prog Cardiovasc Dis. (2009) 52:46–60. 10.1016/j.pcad.2009.05.005 19615493

[30] Tian J Yuan Y Shen M Zhang X He M Guo H. Association of resting heart rate and its change with incident cardiovascular events in the middle-aged and older Chinese. Sci Rep. (2019) 9:6556. 10.1038/s41598-019-43045-5 31024039 PMC6484081

[31] Ferrari R Fox K. Heart rate reduction in coronary artery disease and heart failure. Nat Rev Cardiol. (2016) 13:493–501. 10.1038/nrcardio.2016.84 27226153

[32] Kim H Lee S Ha E Kwon SH Jeon JS Noh H. Age and sex specific target of blood pressure for the prevention of cardiovascular event among the treatment naive hypertensive patients. Sci Rep. (2020) 10:21538. 10.1038/s41598-020-78641-3 33299061 PMC7726552

[33] Santosa A Zhang Y Weinehall L Zhao G Wang N Zhao Q. Gender differences and determinants of prevalence, awareness, treatment and control of hypertension among adults in China and Sweden. BMC Public Health. (2020) 20:1763. 10.1186/s12889-020-09862-4 33228600 PMC7685617

[34] Cai X Zhang Y Li M Wu JH Mai L Li J. Association between prediabetes and risk of all cause mortality and cardiovascular disease: updated meta-analysis. BMJ. (2020) 370:m2297. 10.1136/bmj.m2297 32669282 PMC7362233

[35] Cai X Liu X Sun L He Y Zheng S Zhang Y. Prediabetes and the risk of heart failure: a meta-analysis. Diabetes Obes Metab. (2021) 23:1746–53. 10.1111/dom.14388 33769672

[36] Mai L Wen W Qiu M Liu X Sun L Zheng H. Association between prediabetes and adverse outcomes in heart failure. Diabetes Obes Metab. (2021) 23:2476–83. 10.1111/dom.14490 34227220

[37] Vasan RS Larson MG Leip EP Kannel WB Levy D. Assessment of frequency of progression to hypertension in non-hypertensive participants in the Framingham heart study: a cohort study. Lancet. (2001) 358:1682–6. 10.1016/S0140-6736(01)06710-1 11728544

[38] Lewington S Clarke R Qizilbash N Peto R Collins R Prospective studies collaboration. Age-specific relevance of usual blood pressure to vascular mortality: a meta-analysis of individual data for one million adults in 61 prospective studies. Lancet. (2002) 360:1903–13. 10.1016/S0140-6736(02)11911-8 12493255

